# Efficacy of a novel large-cell Niti-S stent with a slim delivery system for hilar biliary obstruction: a preliminary study

**DOI:** 10.1080/07853890.2022.2056631

**Published:** 2022-04-12

**Authors:** Mitsuru Sugimoto, Tadayuki Takagi, Rei Suzuki, Naoki Konno, Hiroyuki Asama, Yuki Sato, Hiroki Irie, Yuto Ishizaki, Hidenobu Akatsuka, Jun Nakamura, Mika Takasumi, Minami Hashimoto, Tsunetaka Kato, Ryoichiro Kobashi, Takumi Yanagita, Shigeru Marubashi, Takuto Hikichi, Hiromasa Ohira

**Affiliations:** a Department of Gastroenterology, School of Medicine, Fukushima Medical University, Fukushima, Japan; b Department of Gastroenterology, Saiseikai Fukushima General Hospital, Fukushima, Japan; c Department of Gastroenterology, Fukushima Red Cross Hospital, Fukushima, Japan; d Department of Endoscopy, Fukushima Medical University Hospital, Fukushima, Japan; e Department of Hepato-Biliary-Pancreatic and Transplant Surgery, School of Medicine, Fukushima Medical University, Fukushima, Japan

**Keywords:** Large-cell stent, malignant hilar biliary obstruction, slim delivery

## Abstract

**Objectives:**

The large-cell Niti-S stent is useful for multiple stenting in patients with malignant hilar biliary obstruction (MHBO). Recently, a novel uncovered self-expandable metallic stent (USEMS) (a Niti-S large-cell SR slim delivery system) was developed. In this study, we aimed to evaluate the efficacy of this USEMS slim delivery system in MHBO patients.

**Materials and methods:**

Outcomes related to USEMS placement, the clinical course, and the period to recurrent biliary obstruction (RBO) were evaluated in MHBO patients who received multiple USEMSs with the Niti-S large-cell SR slim delivery system.

**Results:**

Twenty-two MHBO patients underwent the placement of multiple USEMSs, including the novel slim-delivery stent. Six patients had a past history of upper gastrointestinal reconstruction (Billroth I: 1, Billroth II: 4, Roux-en-Y: 1). The number of USEMSs placed in each patient was 2-6. Three procedures were reinterventions. The new slim delivery system was placed as the first stent in ten patients and as an additional stent in the remaining patients. Seven patients were drained using only Niti-S large-cell SR slim delivery stents. The technical and clinical success rates were both 100%.

**Conclusions:**

Placing multiple USEMSs in patients with a past history of abdominal surgery or in reintervention is difficult. Although difficult cases were included in this study, stent-in-stent placement with the novel Niti-S large-cell SR slim delivery system was useful in treating MHBO patients. In addition, this novel stent might be the first choice for MHBO patients.

**KEY MESSAGES**
- Endoscopic multistenting for MHBO is challenging. In addition, reintervention or multistenting for MHBO patients with a past history of abdominal surgery becomes more difficult.
- The novel Niti-S large-cell SR slim delivery USEMS is useful as an additional stent because the delivery system is thin and suitable for a 0.025 guidewire. In addition, the novel stent is of the braided type and has a large mesh. Therefore, the novel stent is expected to have strong radial force and can be used as the first SEMS.
- The Niti-S large-cell SR slim delivery stent is long enough to be used in patients with upper gastrointestinal reconstruction. Although this study included patients with reintervention or a past history of upper gastrointestinal reconstruction, the technical success rate of multiple stenting for MHBO patients was 100%. The slim-delivery stent might overcome several difficulties of endoscopic multistenting.

## Introduction

Endoscopic stenting is the first choice for biliary drainage in patients with unresectable malignant hilar biliary obstruction (MHBO). The patency of uncovered self-expandable metallic stents (USEMSs) has been reported to be longer than that of plastic stents (PSs) for MHBO [1,2]. However, whether unilateral or multilateral biliary drainage should be performed is under discussion [3,4], and patients who need multilateral drainage definitely exist (for example, those with cholangitis due to blocked hepatic ducts or liver abscesses) [5]. However, endoscopic multilateral USEMS insertion is technically challenging.

For multilateral drainage in MHBO patients, USEMSs with a large cell size have been reported to be effective [6]. Large cells are advantageous for the placement of additional USEMSs and reintervention. Furthermore, a large-cell USEMS can exert sufficient radial force through the use of a thick nitinol wire.

Recently, a novel large-cell USEMS with a slim delivery system was developed. In this study, we investigated the efficacy of this novel USEMS for drainage in patients with MHBO.

## Materials and methods

### Patients and ethics

MHBO patients who underwent placement of the novel large-cell USEMS between October 2019 and February 2022 were enrolled in this study. Endoscopic retrograde cholangiopancreatography (ERCP) was performed if elevated serum hepatic enzyme and bilirubin levels were observed with biliary obstruction by CT. Endoscopic USEMS insertion was performed at three general hospitals in Japan. The requirement for informed consent was waived because this was a retrospective study using anonymized clinical data. All patients agreed to undergo the clinical examination and treatment by providing written consent. The details of the study can be found on the homepage of Fukushima Medical University. All experimental protocols involving human data were performed in accordance with the Declaration of Helsinki. This study was approved by the Institutional Review Board of Fukushima Medical University (approval number: 2453).

### The novel large-cell USEMS with a slim delivery system

**Figure 1.**
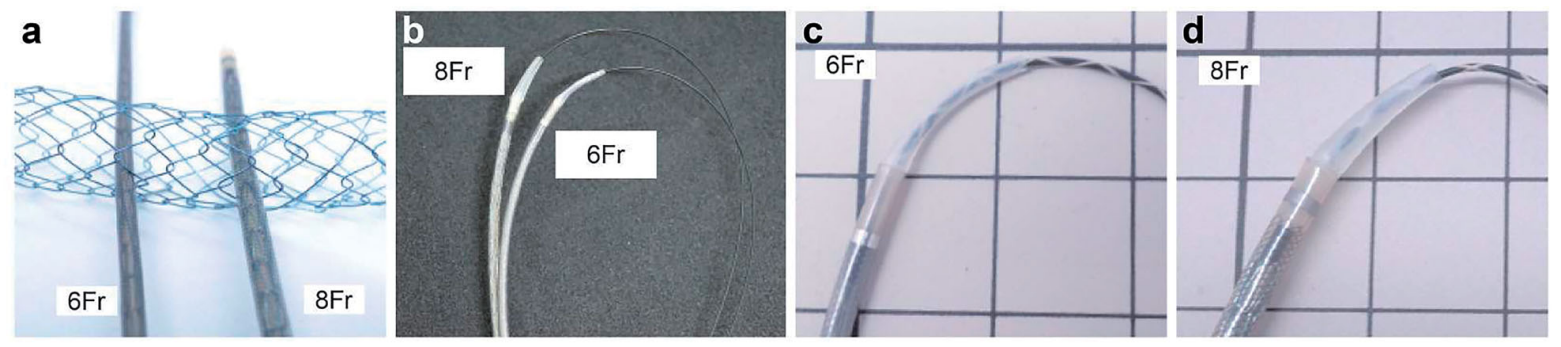
The large-cell Niti-S slim-delivery stent (6 Fr) and the conventional large-cell Niti-S stent (8 Fr). (a) The new slim-delivery Niti-S stent is thinner than the conventional large-cell Niti-S stent. (b) The new slim-delivery Niti-S stent has better trackability for a guidewire than the conventional large-cell Niti-S stent. (c, d) The 6 Fr delivery system has a smaller step between the delivery system and 0.025 guidewire than the 8 Fr delivery system.

The newly designed USEMS used in this study was the Niti-S large-cell SR slim delivery system (Taewoong Medical, Gyeoenggi-do, Korea), as shown in Figure 1 (image provided by Century Medical, Tokyo, Japan). The new USEMS has a 6 Fr delivery system. In contrast, a conventional USEMS (large-cell Niti-S uncovered D-type metallic stent (Taewoong Medical)) has an 8 Fr delivery system (Figure 1(a)). The new slim delivery system has good trackability for a 0.025 guidewire (Figure 1(b)). At the tip of the new delivery system, the step between the 0.025 guidewire and the delivery system is smaller than that at the tip of the conventional 8 Fr delivery system (Figure 1(c,d)).

**Figure 2.**
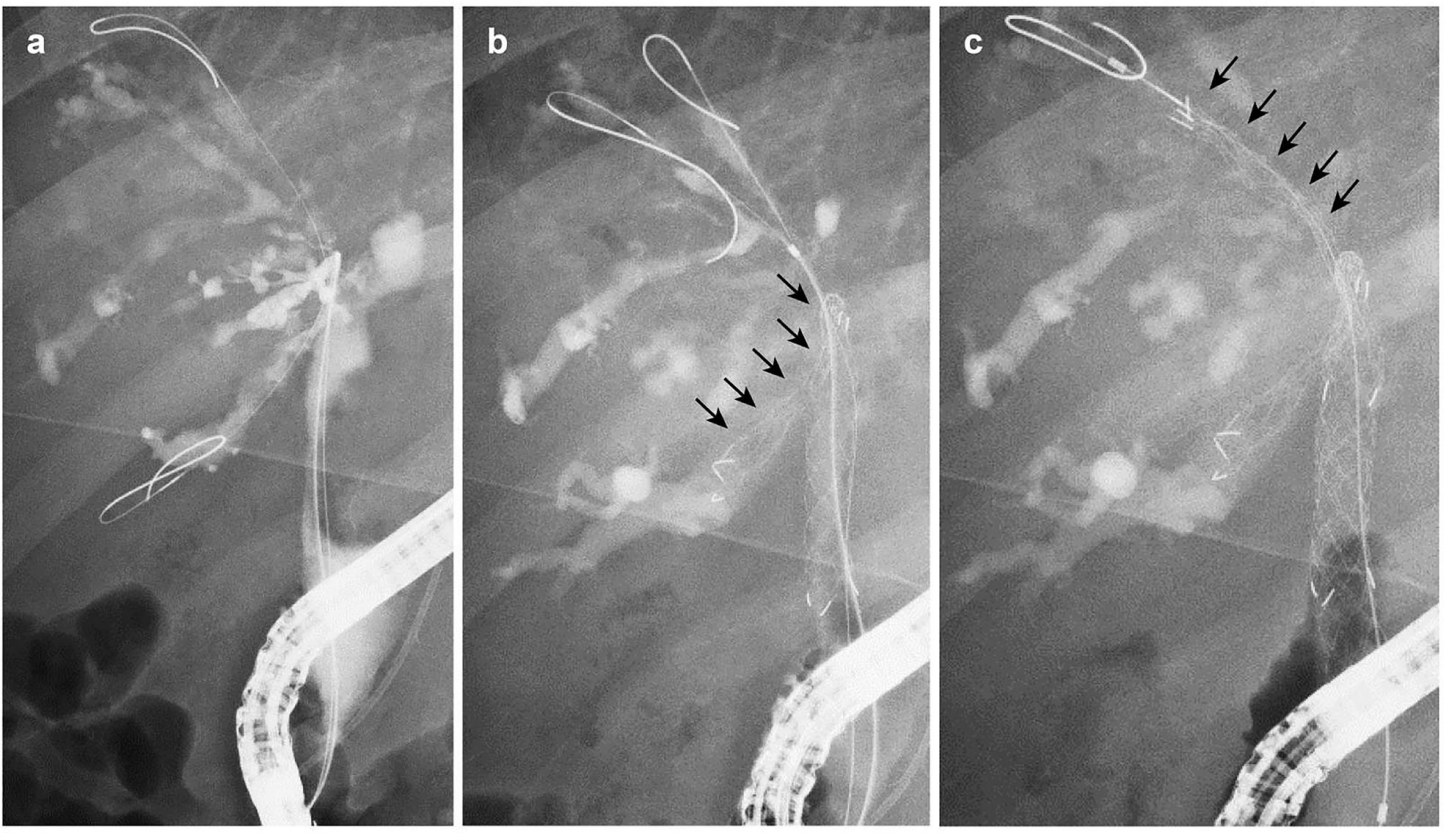
A case of MHBO with primary sclerosing cholangitis. (a) Bismuth IIIa MHBO was observed by ERCP. (b) A 10 mm × 10 cm large-cell Niti-S slim-delivery stent was placed in B6. (c) After the other guidewire was placed in B5, the new slim-delivery USEMS was placed along the guidewire. MHBO: malignant hilar biliary obstruction; ERCP: endoscopic retrograde cholangiopancreatography; USEMS: uncovered self-expandable metallic stent.

### Endoscopic retrograde cholangiopancreatography (ERCP) procedure

The patients were sufficiently sedated with midazolam and pentazocine before endoscope insertion. After the endoscope reached the Vater papilla or bile duct anastomotic site, biliary cannulation was initiated. The state of the biliary stricture was confirmed by cholangiography (Figure 2(a)); then, a guidewire was advanced to an objective biliary branch, and the first USEMS was inserted. If the MHBO patient had an untreated Vater papilla, endoscopic sphincterotomy (EST) was performed after cholangiography. Another guidewire was advanced to another biliary branch through the mesh of the first USEMS (Figure 2(b)). Dilation of the mesh was performed as needed, and a second USEMS was inserted (Figure 2(c)). For multiple USEMS insertions, the steps after guidewire insertion were repeated as needed.

A fixed method for multiple USEMS insertions has not been established at our hospital. However, in most such procedures in the present study, the first USEMS was inserted in the left hepatic duct, and the second USEMS was inserted in the right hepatic duct. When multiple right hepatic duct drainage procedures were needed, the USEMSs were usually placed in order of descending angle between the common bile ducts. All USEMSs were placed using the stent-in-stent method. A conventional Niti-S large-cell stent was used for as many patients as possible because the thick nitinol wire was expected to enable a strong radial force and larger mesh. When balloon enteroscopy was used or when the targeted biliary duct was thin (for example, primary sclerosing cholangitis), the new Niti-S large-cell SR slim delivery stent was used as the first SEMS. A laser-cut SEMS (Zilver 635, COOK Medical Japan, Tokyo, Japan) was used as the first SEMS in a patient because the laser cut SEMS was also expected to have a large mesh. The laser-cut SEMS was also used as the secondary SEMS in one patient because a Niti-S large-cell SR slim delivery stent of the same length had already been used as the first SEMS, and the Niti-S large-cell SR slim delivery stent was out of stock. Other SEMSs were randomly selected for use at other previous hospitals.

The endoscope used in this study was a JF-260V or SIF-H290S device (Olympus, Tokyo, Japan). The ERCP catheter was a Tandem XL (Boston Scientific Japan, Tokyo, Japan) or MTW tapered catheter (MTW Endoskopie, Wesel, Germany). VisiGlide2 EndoSelector guidewires (Boston Scientific Japan, Tokyo, Japan) were employed. The dilation devices used in this study were a 6 mm REN biliary dilation catheter (Kaneka Corporation, Tokyo, Japan) or ES dilator DC7F180S (Zeon Medical Co., Tokyo, Japan). The USEMSs that the patients had previously received were the 10 mm × 6 cm Zilver 635 stent (COOK Medical Japan), 10 mm x 6 cm ZEO stent (Zeon Medical Co.), or 10 mm × 8 cm or 10 cm large-cell Niti-S D type stent (Taewoong Medical). The USEMSs used in this study were the 10 mm × 8 cm or 10 cm large-cell Niti-S D-type stent (Taewoong Medical), 10 mm × 6 cm Zilver 635 stent (COOK Medical Japan, Tokyo, Japan), and 8 mm × 8 cm or 10 cm, 10 mm × 6 cm or 8 cm Niti-S large-cell SR slim delivery stent (Taewoong Medical).

### Examination items

The primary outcome of this study was the technical success rate. Patient characteristics (age, sex, primary lesion site, past history of abdominal surgery, diagnoses, serum data (alanine aminotransferase (ALT), total bilirubin (TB)), and Bismuth classification), outcomes of endoscopic USEMS placement (procedural time, number of sessions, number of USEMSs, identity of the hepatic ducts in which the USEMSs were placed, number of placed USEMSs, dilation device usage, clinical success), clinical course after USEMS placement (adverse events, chemotherapy after USEMS placement, USEMS dysfunction, cause of USEMS dysfunction, period to recurrent biliary obstruction (RBO), death, and follow-up period) were also evaluated.

Technical success was defined as the successful placement of multiple USEMSs in the intended biliary ducts with sufficient coverage of the stricture. Clinical success was defined as a 50% decrease in or normalization of hepatobiliary enzymes within 14 days after USEMS placement. Stent dysfunction was defined as elevated hepatobiliary enzyme levels or the appearance of a dilated hepatic duct without pneumobilia on computed tomography (CT) or ultrasound that needed additional endoscopic drainage. The period to RBO was defined as the period from Niti-S large-cell SR slim delivery stent insertion to stent dysfunction. The evaluated adverse events were pancreatitis, bleeding, and perforation. These events were defined according to an article written by Isayama et al. [7] and Cotton et al.’s [8] criteria.

## Results

**Figure 3.**
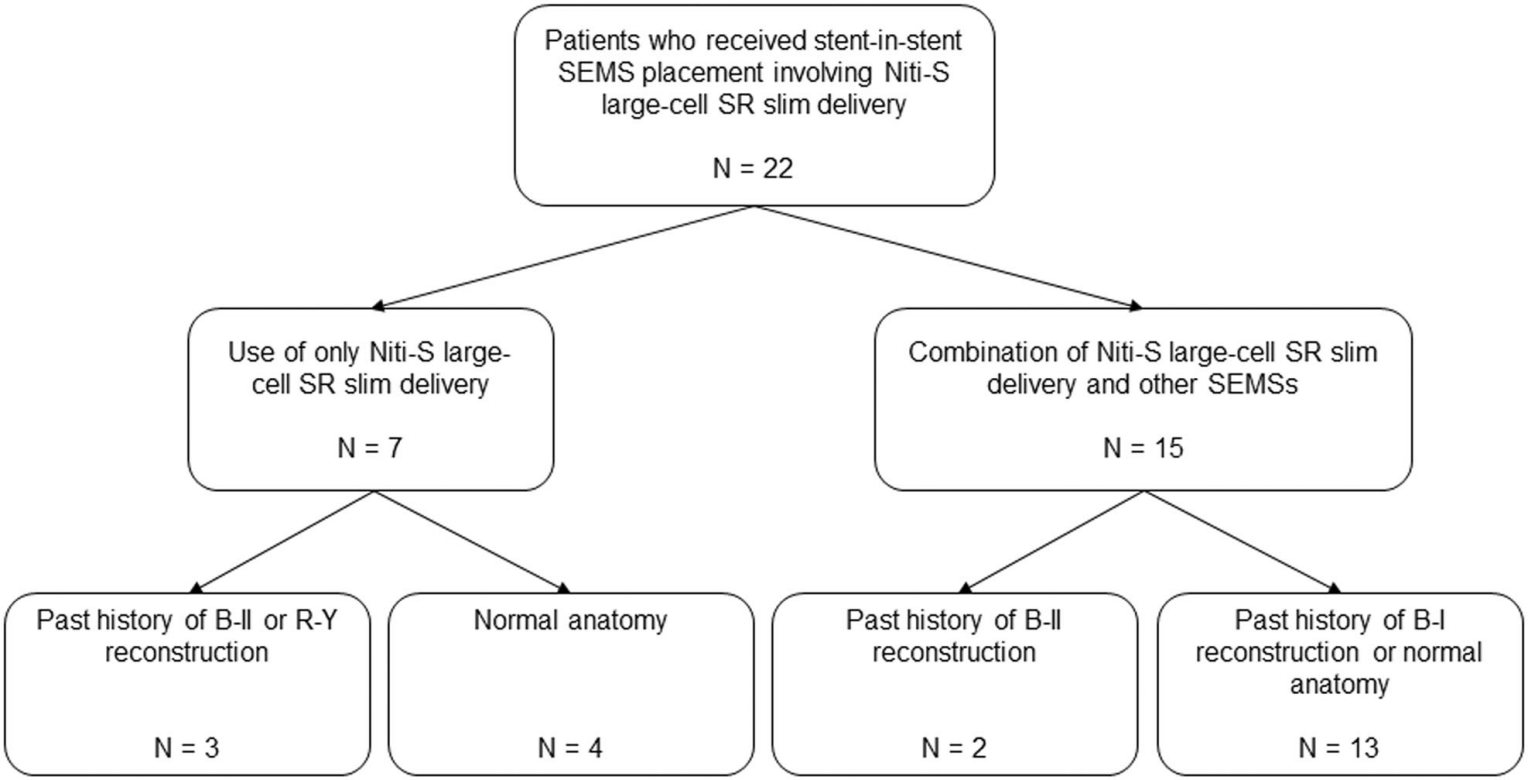
Flow chart of MHBO patients who underwent stent-in-stent SEMS placement involving the Niti-S large-cell SR slim delivery system. SEMS: self-expandable metallic stent; B: Billroth; R-Y: Roux-en-Y.

Twenty-two MHBO patients underwent the placement of multiple USEMSs, including the novel slim-delivery stent (Figure 3, Table 1). Only the Niti-S large-cell SR slim delivery stent was used in 7 patients. Among them, 3 patients had a past history of Billroth (B)-II reconstruction (*n* = 2) or Roux-en-Y (R-Y) reconstruction (*n* = 1), and 4 patients had a normal anatomy. On the other hand, 15 patients were treated with a combination of Niti-S large-cell SR slim delivery and other SEMSs. Among them, two patients had a past history of B-II reconstruction. The other 13 patients had a normal anatomy (*n* = 12) or a past history of B-I reconstruction (*n* = 1). Among the 22 patients, 11 were diagnosed by biliary biopsy, and 5 were diagnosed by biliary juice cytology. Three patients with a past history of surgery were observed to have evident tumour recurrence by CT. Another two patients were diagnosed by positron emission tomography. The other patient with liver cancer was diagnosed by dynamic CT. The median age of the patients was 74.0 (51–90) years. The primary lesion sites were as follows: bile duct (14 patients), gallbladder (4 patients), stomach (2 patients), liver (1 patient), and colon (1 patient). The median ALT level was 89 (11–377) U/L. The median TB level was 2.6 (0.3–18.8) mg/dL. The Bismuth classification distribution was as follows: II (5 patients), IIIa (4 patients), IIIb (4 patients), and IV (9 patients).

**Table 1.** Patient characteristics.

No.	Age	Sex	Primary lesion site	Past history of upper gastrointestinal reconstruction	ALT (U/L)	TB (mg/dL)	Bismuth classification
*Using only Niti-S large-cell SR slim delivery*							
Past history of B-II or R-Y reconstruction							
1	77	F	Bile duct	Pancreaticoduodenectomy	110	11.5	IV
				B-II reconstruction			
2	75	F	Gallbladder	Pancreaticoduodenectomy	26	0.7	IV
				B-II reconstruction			
3	69	F	Bile duct	Extended left hepatectomy	11	0.3	IV
				R-Y reconstruction			
Normal anatomy							
4	72	M	Bile duct	No	100	8.5	IIIa
5	84	F	Colon	No	37	14.5	IIIa
6	82	F	Bile duct	No	40	2	IIIb
7	78	F	Bile duct	No	85	16.0	IV
*Combination of Niti-S large-cell SR slim delivery and conventional SEMSs*							
Past history of B-II reconstruction							
8	72	M	Stomach	Gastrojejunostomy	377	0.7	II
				B-II reconstruction			
9	81	M	Bile duct	Pancreaticoduodenectomy	30	0.7	IIIb
				B-II reconstruction			
Normal anatomy or a past history of B-I reconstruction							
10	90	M	Bile duct	Gastrectomy	130	3.0	II
				B-I reconstruction			
11	70	M	Bile duct	No	22	1.5	IV
12	68	M	Gallbladder	No	35	1.5	IV
13	71	M	Bile duct	No	102	2.2	IIIb
14	51	M	Bile duct	No	112	17.1	IV
15	73	M	Bile duct	No	61	2.0	IIIa
16	51	M	Stomach	No	110	8.1	IV
17	84	M	Gallbladder	No	16	0.6	IIIa
18	87	M	Bile duct	No	46	18.8	II
19	82	F	Bile duct	No	303	0.7	II
20	77	M	Liver	No	189	11.3	IIIb
21	73	M	Bile duct	No	223	5.0	IV
22	68	F	Gallbladder	No	93	12.2	II

ALT: alanine aminotransferase; TB: total bilirubin; M: male; F: female; B: Billroth; R-Y: Roux-en-Y.

The outcomes of endoscopic USEMS placement are shown in Table 2. The procedural time was 70 (26–137) minutes (Among patients with only Niti-S large-cell SR slim delivery stents, those with R-Y or B-II reconstruction had a procedural time of 99 (47–101) minutes, and those with a normal anatomy had a procedural time of 38.5 (37–137) minutes. Among those with a combination of Niti-S large-cell SR slim delivery stent and other SEMSs, those with B-II reconstruction had a procedural time of 70 and 89 min, and those with B-I reconstruction or a normal anatomy had a procedural time of 70 (26–117) minutes). In three patients, the procedure required two sessions (patient no 12, 14, 15). Among them, two patients required reintervention (patient no 14, 15). The number of placed USEMSs in each patient was 2–6. All patients who underwent placement of more than three SEMSs underwent stent-in-stent placement with a combination of Niti-S large-cell SR slim delivery stent and convetional SEMSs, and had a normal anatomy or B-I reconstruction (patient no. 10–22). The maximum number of hepatic ducts in which USEMSs were inserted was four (patient no. 12). Although patient no. 14 received six USEMSs, three were reinterventions. The USEMS insertion in patient no. 15 was also a reintervention. In ten patients, the new slim delivery system was used to place the first stent. In the other patients, the Niti-S large-cell SR slim delivery stent was used as an additional stent. In six patients, dilation devices were used (1 patient with only a Niti-S large-cell SR slim delivery stent and a normal anatomy and 5 patients with a combination of Niti-S large-cell SR slim delivery and B-I reconstruction or a normal anatomy). The technical and clinical success rates were both 100%, regardless of a past history of upper gastrointestinal reconstruction or reintervention.

**Table 2.** Outcomes of endoscopic USEMS placement.

No.	Procedural time (min)	Session	No. of USEMSs	Hepatic duct placed USEMS	Placed USEMSs	Dilation device usage	Technical success	Clinical success
*Using only Niti-S large-cell SR slim delivery*								
Past history of B-II or R-Y reconstruction								
1	101	1	2	B3, RAD	1. Niti-S slim delivery 10 mm 6 cm	No	Yes	Yes
					2. Niti-S slim delivery 10 mm 6 cm			
2	47	1	2	B3, B8	1. Niti-S slim delivery 10 mm 6 cm	No	Yes	Yes
					2. Niti-S slim delivery 10 mm 6 cm			
3	99	1	2	B8, B6	1. Niti-S slim delivery 10 mm 6 cm	No	Yes	Yes
					2. Niti-S slim delivery 10 mm 6 cm			
Normal anatomy								
4	137	1	2	B6, 5	1. Niti-S slim delivery 10 mm 10 cm	Yes	Yes	Yes
					2. Niti-S slim delivery 10 mm 10 cm			
5	37	1	2	L, B6	1. Niti-S slim delivery 10 mm 8 cm	No	Yes	Yes
					2. Niti-S slim delivery 8 mm 8 cm			
6	63	1	2	B2, B8	1. Niti-S slim delivery 8 mm 10 cm	No	Yes	Yes
					2. Niti-S slim delivery 8 mm 10 cm			
7	40	1	2	B3, B8	1. Niti-S slim delivery 8 mm 10 cm	No	Yes	Yes
					2. Niti-S slim delivery 8 mm 10 cm			
*Combination of Niti-S large-cell SR slim delivery and conventional SEMSs*								
Past history of B-II reconstruction								
8	70	1	2	L, RAD	1. Niti-S large-cell D type 10 mm 10 cm	No	Yes	Yes
					2. Niti-S slim delivery 10 mm 6 cm			
9	98	1	2	R, L	1. Niti-S slim delivery 10 mm 6 cm	No	Yes	Yes
					2. Zilver 635 10 mm 6 cm			
Normal anatomy or past history of B-I reconstruction								
10	89	1	2	L, R	1. Niti-S large-cell D type 10 mm 8 cm	Yes	Yes	Yes
					2. Niti-S slim delivery 10 mm 6 cm			
11	66	1	3	B2, 6, 8	1. Niti-S large-cell D type 10 mm 10 cm	No	Yes	Yes
					2. Niti-S slim delivery 10 mm 8 cm			
					3. Niti-S slim delivery 10 mm 6 cm			
12	106	2	4	L, B6, RAD (first session) B7	1. Niti-S large-cell D type 10 mm 10 cm	Yes (before 3rd USEMS)	Yes	Yes
					2. Niti-S large-cell D type 10 mm 8 cm			
					3. Niti-S slim delivery 10 mm 8 cm			
					4. Niti-S slim delivery 10 mm 6 cm			
13	49	1	2	RPD, RAD	1. Niti-S large-cell D type 10 mm 8 cm	No	Yes	Yes
					2. Niti-S slim delivery 10 mm 8 cm			
14	75	2	6	RPD, B5,8 (previous institution) B8, RPD, B5	1. Zilver 635 10 mm 6 cm	Yes (before fifth and sixth USEMS)	Yes	Yes
					2, 3. ZEO STENT 10 mm 6 cm			
					(previous institution)			
					4. Niti-S large-cell D type 10 mm 10 cm			
					5. Niti-S slim delivery 10 mm 8 cm			
					6. Niti-S slim delivery 10 mm 6 cm			
15	70	2	4	L, B5 (previous session) L, B5	1. Niti-S large-cell D type 10 mm 8 cm	Yes	Yes	Yes
					2. Niti-S large-cell D type 10 mm 8 cm			
					3. Niti-S large-cell D type 10 mm 8 cm			
					4. Niti-S slim delivery 10 mm 8 cm			
16	102	1	2	L, RAD	1. Zilver 635 10 mm 10 cm	No	Yes	Yes
					2. Niti-S slim delivery 10 mm 10 cm			
17	26	1	2	L, R	1. Niti-S large-cell D type 10 mm 8 cm	No	Yes	Yes
					2. Niti-S slim delivery 10 mm 8 cm			
18	43	1	2	L, R	1. Niti-S large-cell D type 10 mm 10 cm	No	Yes	Yes
					2. Niti-S slim delivery 10 mm 8 cm			
19	60	1	2	L, R	1. Niti-S large-cell D type 10 mm 10 cm	Yes	Yes	Yes
					2. Niti-S slim delivery 10 mm 10 cm			
20	60	1	2	B2, B5	1. Niti-S slim delivery 10 mm 8 cm	No	Yes	Yes
					2. Niti-S large-cell D type 10 mm 8 cm			
21	82	1	2	B2, B7	1. Niti-S large-cell D type 10 mm 10 cm	No	Yes	Yes
					2. Niti-S slim delivery 10 mm 8 cm			
22	117	1	2	L, R	1. Niti-S slim delivery 10 mm 8 cm	No	Yes	Yes
					2. Niti-S large-cell D type 10 mm 8 cm			

USEMS: uncovered self-expandable metallic stent; B: Billroth; R-Y: Roux-en-Y; L: left hepatic duct; RAD: right anterior hepatic duct; RPD: right posterior hepatic duct; R: right hepatic duct.

The clinical courses after USEMS placement are shown in Table 3. Adverse events were not observed. In ten patients, chemotherapy was performed after USEMS placement. USEMS dysfunction was observed in three patients (cause of dysfunction: ingrowth 2, overgrowth 1). On the other hand, stent dysfunction was not observed in patients 14 and 15, both of whom underwent reintervention. The median period to RBO was 99.5 (9–402) days. Eighteen patients died. The median follow-up period was 125.5 (9–402) days.

**Table 3.** Clinical course after USEMS placement.

No.	Adverse events	Chemotherapy after USEMS placement	USEMS dysfunction	Cause of USEMS dysfunction	The period to RBO (days)	Live/Dead	Follow-up period (days)
*Using only Niti-S large-cell SR slim delivery*							
Past history of B-II or R-Y reconstruction							
1	No	No	No		63	Dead	63
2	No	Yes	Yes	Ingrowth	100	Live	112
3	No	Yes	No		30	Live	30
Normal anatomy							
4	No	Yes	No		99	Dead	99
5	No	No	No		71	Dead	71
6	No	No	No		28	Dead	28
7	No	No	No		18	Dead	18
*Combination of Niti-S large-cell SR slim delivery and conventional SEMSs*							
Past history of B-II reconstruction							
8	No	Yes	No		169	Dead	169
9	No	No	No		139	Dead	139
Normal anatomy or past history of B-I reconstruction							
10	No	No	No		193	Dead	193
11	No	Yes	No		402	Live	402
12	No	Yes	No		252	Dead	252
13	No	Yes	Yes	Ingrowth	50	Dead	204
14	No	No	No		42	Dead	42
15	No	No	No		107	Dead	107
16	No	No	No		9	Dead	9
17	No	No	No		322	Dead	322
18	No	No	No		81	Dead	81
19	No	No	No		246	Dead	246
20	No	Yes	No		200	Dead	200
21	No	Yes	No		173	Live	173
22	No	Yes	Yes	Overgrowth	56	Dead	208

USEMS: uncovered self-expandable metallic stent; RBO: recurrent biliary obstruction; B: Billroth; R-Y: Roux-en-Y.

## Discussion

In this study, 22 MHBO patients underwent the endoscopic placement of multiple USEMSs, including the Niti-S large-cell SR slim delivery stent. Although five patients with a past history of abdominal surgery other than B-I reconstruction and three reinterventions were included, both the technical and clinical success rates were 100%.

In a recent randomized controlled trial, the technical success rate of bilateral biliary drainage with a USEMS was 90% [4]. With the use of a previously developed 8.5 Fr large-cell Niti-S stent (Taewoong Medical), the technical success rate was 96% [6]. However, ERCP is challenging to perform in patients with surgically altered anatomy. In a report with a large number of patients, the success rate of enteroscope insertion was 71%, and the technical success rate was 63% [9]. In addition, reintervention for stent occlusion after bilateral USEMS placement was difficult. The technical success rate of reintervention with bilateral USEMS placement was reported to be 13% in patients who had previously undergone bilateral USEMS placement [10]. In the present study, the technical and clinical success rates using the slim-delivery USEMS were similar to those of a recent randomized controlled trial and a past report using a previously developed 8.5 Fr large-cell stent [4,6]. In addition, all multiple stenting procedures were successful in patients with a past history of abdominal surgery and in patients with reintervention. Although such difficult cases were included, the technical success rate was 100%. Regarding the reason for the good technical success rate, there is a possibility that the Niti-S large-cell SR slim delivery system overcomes several risk factors for multiple biliary drainage failure.

Few reports have investigated the risk factors for failed placement of multiple USEMSs with the stent-in-stent technique. Kawakubo et al. [11] identified metastatic diseases as a risk factor for the failure of stent-in-stent USEMS placement. In contrast, a large mesh and thin delivery system were favourable factors. Sugimoto et al. [12] used large-mesh USEMSs and investigated the risk factors for the failure of stent-in-stent USEMS placement in patients. They reported that an angle greater than 49.7 degrees between the first USEMS and second hepatic duct was a risk factor for the failure of stent-in-stent insertion. In the present study, ten patients showed an angle greater than 49.7 degrees between the first USEMS and the biliary branch, where the second USEMS needed to be placed. In nine of the ten patients with large angles, the Niti-S large-cell SR slim delivery stent was successfully placed as the second stent. Three MHBO patients in this study had metastatic disease; however, the successful placement of multiple USEMSs was achieved by using the Niti-S large-cell SR slim delivery system. These satisfactory results may be because the Niti-S large-cell SR slim delivery stent has features favourable for stent-in-stent placement, including a large mesh and thin delivery system. A large mesh facilitates the placement of additional USEMSs, and a thin delivery system facilitates passage through the mesh. In fact, in most cases in the present study, USEMS placement using the Niti-S large-cell SR slim delivery stent was achieved without the use of any dilation devices. Recently, several SEMSs with slim delivery systems have been developed (Table 4) [13–15]. Among them, the Niti-S large-cell SR slim delivery system has shown several merits. First, a braided-type SEMS has a stronger extended force than a laser-type SEMS. Second, a longer effective length of the delivery system is suitable for stenting in MHBO patients with a past history of abdominal surgery [16,17]. Third, the Niti-S large-cell SR slim delivery is suitable for a 0.025 guidewire, and there is almost no step between the guidewire and the tip of the delivery system. In addition to having a large-sized cell, only the Niti-S large-cell SR slim delivery stent meets all three conditions. Because of the small sample size, it is difficult to conclude that Niti-S large-cell SR slim delivery is useful for patients with a past history of abdominal surgery. However, for patients undergoing SEMS placement with a past history of upper gastrointestinal surgery, the Niti-S large-cell SR slim delivery system might be suitable due to the SEMS design and delivery system (Table 4). The other SEMSs with sufficiently long delivery systems were the laser type or suitable for a 0.035 guidewire. As described above, a laser-type SEMS has less radial force than a braided-type SEMS. Therefore, the Niti-S large-cell SR slim delivery system might become the first choice for patients with B-II or R-Y reconstruction.

**Table 4.** USEMSs with a slim delivery system.

USEMS	Type	Diameter of delivery system (Fr)	Effective length (cm)	Suitable guidewire
Niti-S large-cell slim delivery (Taewoong Medical, Gyeoenggi-do, Korea)	Braided	6.0	196	0.025
EGIS Braided 6 (Sumitomo Bakelite, Tokyo, Japan)	Braided	6.0	180	0.025
HANAROSTENT benefit (Boston Scientific Japan, Tokyo, Japan)	Braided	5.9	180	0.025
Epic biliary stent (Boston Scientific Japan)	Laser	6.0	220	0.035
ZILVER635 (Cook Medical Japan, Tokyo, Japan)	Laser	6.0	200	0.035
BILERUSH SELECTIVE (PIOLAX, Yokohama, Japan)	Laser	5.7	190	0.035
ZEOSTENT V (ZEON MEDICAL, Tokyo, Japan)	Laser	5.4	200	0.025
YABUSAME (KANEKA, Tokyo, Japan)	Laser	5.4	200	0.025

USEMS: uncovered self-expandable metallic stent.

On the other hand, one concern of adopting a thin delivery system is earlier SEMS dysfunction. The patency rate 150 days after bilateral conventional SEMS placement was reported to be 50 (6/12)–60.9 (39/64)% [18,19]. In most patients in this study, conventional large-cell stents were used because a strong radial force is expected with a thick nitinol wire [18]. However, stent dysfunction was observed in only two of the ten patients who received a Niti-S large-cell SR slim delivery stent as the first stent (patients 2, 22). In addition, in these patients, a second USEMS was successfully placed. The hepatic ducts of patient 4 were hard and thin, with primary sclerosing cholangitis. The Niti-S large-cell SR slim delivery stent was also suitable for placement in the thin biliary duct. Because of these factors, the Niti-S large-cell SR slim delivery stent is expected to be used as the first stent.

This study has several limitations. First, this was a retrospective study with a small number of patients. However, according to a past study in our institution, the technical success rate of stent-in-stent SEMS placement for MHBO patients was 75.4% when using conventional SEMSs [12]. In this study, the technical success rate was 100%. Therefore, a total of 21 patients were necessary to achieve an α error of 5% and a *β* value of 0.2. When the technical success rate was determined as the main outcome, a sufficient number of patients participated in this study. In the future, a prospective study should be performed to confirm the results. Second, not all stenting was performed with only the Niti-S large-cell SR slim delivery system. However, this stent was used as the first stent and for the placement of multiple stents or for reintervention. The results of this study show that the USEMS and slim delivery system can be used to treat several types of MHBO.

## Conclusions

Multiple stent-in-stent SEMSs placed using the Niti-S large-cell SR slim delivery system was useful for treating MHBO. The Niti-S large-cell SR slim delivery stent was suitable for use as an additional SEMS because of the good trackability for a 0.025 guidewire and the smaller step between the 0.025 guidewire and the delivery system. On the other hand, the Niti-S large-cell SR slim delivery stent was expected to be used as the first SEMS because of the large mesh. In addition, the Niti-S large-cell SR slim delivery system might become the first choice in patients with a past history of upper gastrointestinal reconstruction because of the stent design, such as the long delivery system and braided type.

## Data Availability

The datasets generated and/or analyzed during the current study are available from the corresponding author upon reasonable request.
